# SETDB-1: A Potential Epigenetic Regulator in Breast Cancer Metastasis

**DOI:** 10.3390/cancers11081143

**Published:** 2019-08-09

**Authors:** Jacob Batham, Pek Siew Lim, Sudha Rao

**Affiliations:** Melanie Swan Memorial Translational Centre, Faculty of Sci-Tech, University of Canberra, Bruce ACT 2617, Australia

**Keywords:** breast cancer, cancer stem cells, therapy, resistance, recurrence, SETDB1, epigenetics

## Abstract

The full epigenetic repertoire governing breast cancer metastasis is not completely understood. Here, we discuss the histone methyltransferase SET Domain Bifurcated Histone Lysine Methyltransferase 1 (SETDB1) and its role in breast cancer metastasis. SETDB1 serves as an exemplar of the difficulties faced when developing therapies that not only specifically target cancer cells but also the more elusive and aggressive stem cells that contribute to metastasis via epithelial-to-mesenchymal transition and confer resistance to therapies.

## 1. Introduction

Breast cancer is the most common cancer in women over the age of 50 [1], with an average of 43 women diagnosed per day, accounting for 15.5% of cancer-related deaths in Australia. While the five-year survival rate for locally invasive breast cancer is >90%, the five-year survival rate after metastases develop is approximately 32% [2]. In an effort to bridge this gap, resources and efforts are increasingly focusing on understanding the mechanisms underpinning metastatic transition and the development of therapeutic resistance.

The prevalence of cancer and its resistance to therapies is due to the presence of a group of cells called cancer stem cells (CSCs). CSCs are a subpopulation of cells with “stem-like” properties, present within a tumor that has the ability to self-renew, and they are important in tumor initiation, formation, and recurrence [3]. The self-renewal ability of a CSC enables it to produce multiple progeny such as tumor progenitor cells, as well as a replica of itself. A subset of these cells can differentiate and form solid tumors while the CSCs remain quiescent. While chemotherapy is able to eradicate differentiated cancer cells, it is not effective against CSCs, which are able to metastasize and propagate tumors causing tumor recurrence and therapy resistance. Furthermore, CSCs also undergo dynamic changes in phenotype through epithelial-to-mesenchymal transition (EMT), a key mechanism in cancer cell metastasis [4].

EMT is an evolutionarily conserved process that underpins tissue remodeling events during embryogenesis, where normal epithelial cells acquire traits which allow them to transition into motile cells [5]. This process is a complex, multi-faceted program involving multiple changes in cell properties and the expression of characteristic tissue markers. EMT is a key process in tumor metastasis because EMT also confers the property of motility [6]. The key hallmarks facilitating EMT progression are the concurrent loss of principal epithelial surface makers, such as E-cadherin (CDH1), and the gain of aberrative mesenchymal surface markers, such as vimentin and N-cadherin (CDH2). This metamorphic event permits cancer stem cells to break away and become motile or sometimes become quiescent [7].

Breast cancer stem cells (BCSCs) are characterized mainly by CD44+/CD24− (cluster of differentiation 44+/cluster of differentiation 24−), with other surface markers such as cluster of differentiation 133 (CD133), epithelial cell adhesion molecule (EpCAM), nestin, ganglioside GD2, CD49f, C-X-C chemokine receptor type 4 (CXCR4), C-X-C Motif Chemokine Ligand 1 (CXCL1), Hydroxymethylglutaryl-CoA synthase (HMGCS), cluster of differentiation 166 (CD166), cluster of differentiation 47 (CD47), Aldehyde dehydrogenase 1 (ALDH1), and ATP-binding cassette super-family G member 2 (ABCG2) identified to be associated with BCSCs as well [8,9]. Another top marker for BCSCs is aldehyde dehydrogenase 1 (ALDH1), an intracellular enzyme that oxidizes aldehydes and retinol. This marker is linked with poor clinical outcomes, as tumors with this marker are resistant to treatment and display a very aggressive clinical phenotype [10]. Different breast cancer subtypes display different BCSC populations, which express different surface markers. Furthermore, not all of these markers are exclusive to BCSCs and are differentially expressed in different cells of the body [11]. The lack of clear characterization of surface markers that can be therapeutically targeted poses a challenge in developing drugs that can directly ablate BCSCs.

At a molecular level, cancers are caused by cumulative molecular aberrations that destabilize the normally tightly regulated process of gene expression [12]. Central to this, the epigenetic regulation of chromatin plays a critical role in establishing and maintaining cellular integrity. Chromatin regulation controls the physical access of various molecules, including enhancers and repressors to DNA to regulate gene expression [13], examples being chromatin-binding factors that bind chromatin or DNA directly or indirectly through protein–protein complex formation [14]. Throughout the genome, specific DNA regions house regulatory genes that maintain cellular homeostasis [15]. Some of these genes are checkpoint inhibitors or tumor suppressors that regulate the cell cycle and mitosis; thus, they are directly involved in aberrant proliferation in cancer [7]. These genes must meet specific criteria in order to progress the cell cycle and, thus, can contribute to uncontrolled proliferation when dysregulated [12]. Dysregulated chromatin modification can have severe consequences, typically genome destabilization, disease development, and most commonly carcinogenesis. There is, therefore, intense interest in the area of chromatin/histone regulation and the impact of the epigenome on gene expression [13].

The dynamic nature of epigenetic regulation lends itself to regulating the process of differentiation and dedifferentiation, which is highly plastic in nature [16]. In particular, within the EMT model, the regulation of these processes is highly dependent on epigenetic instructions that modify the histone template, which in turn generate global expression fluctuations in EMT transitions. This regulatory control is a tightly regulated process that is considered to be a hallmark of cancer dissemination. The functional alteration between EMT-inducing transcription factors and the histone template provides information on how cancer cells acquire stemness, as well as their tumorigenic properties [17].

The histone methyltransferase SET Domain Bifurcated Histone Lysine Methyltransferase 1 (SETDB1) is responsible for the di- and tri-methylation of histone H3 lysine 9 (H3K9) in euchromatic regions (“open” chromatin) to promote gene silencing through heterochromatin (“closed” chromatin) formation [18]. SETDB1 was shown to regulate and influence normal cellular homeostasis, while it is also implicated in breast cancer formation [19]; however, its role in metastasis is rather less well understood. The overexpression of SETDB1 sustains proliferative signaling and metastatic acquisition by modulating epigenetic marks associated with aberrative EMT-mediated gene expression. SETDB1 is known to contribute to the EMT process in breast cancer by interacting with the SMAD/ transforming growth factor-beta (TGF-B) regulatory pathway that influences EMT-inducing transcription factors such as zinc finger protein SNAI1 (Snail-1). The SMADs comprise a family of intracellular proteins that act as signal transducers for TGF-B signaling and are known to significantly impact the regulation of cell development and cell growth in normal cells, as well as in breast cancer development [20]. The overexpression of SETDB1 interacts with these pathways in the breast cancer model and significantly impacts the induction of EMT transcription factors that are associated with metastatic development. However, there are few data illustrating the relationship of SETDB1 to the EMT processes in mouse tumor models and patient tumor tissue samples from metastatic breast cancer patients. While this is the case, recently, the experimental use of digital fluorescence confocal microscopy in triple-negative breast cancer patients illustrated the potential use of this technology for the identification of the crosstalk between epigenetic modifications and the resulting interaction with the EMT-associated transcription factors that are interrelated with metastatic progression [21]. An experimental approach to how SETDB1 interacts with EMT-inducing transcription factors in human breast cancers may serve as a valid candidate for further investigation.

The structural and functional dynamic of the SETDB1 enzyme in cell dysregulation is not well characterized. Therefore, in this review, we illustrate SETDB1 epigenetic activity in breast cancer tissues to potentially influence breast cancer metastasis through the direct and indirect actions on EMT programs via methylation of the histone template, as well as via non-histone substrates. Here, we review the biology of SETDB1 and its interacting partners and explain its role in breast cancer metastasis and therapeutic resistance. We also outline how targeting SETDB1 may be a valuable therapeutic intervention in breast cancer patients.

## 2. Epigenetic Regulation Leads to Cellular Differentiation by Chromatin Modification

While all nucleated cells contain the same genome, differentiation into specific cell types is controlled by gene expression, which is heavily influenced by epigenetic regulation. Although the DNA sequence and transcription factors represent the basic transcriptional machinery, access to these areas is controlled by epigenetic regulators [13]. This allows one line of genetic code to be used in different ways in different cell and tissue contexts. The human genome is condensed into DNA, which is then further condensed into chromosomes formed of repeating, tightly packed units called nucleosomes. Nucleosomes consist of paired histone proteins (H2A, H2B, H3, and H4) that form an octamer surrounding the DNA, helping it compact and form chromatin [13]. Chromatin exists in two conformational states, euchromatin and heterochromatin; euchromatin is “open” DNA, which permits active transcription, while heterochromatin is “closed” DNA, which represses transcription. These open and closed states are influenced by modifications including but not limited to DNA methylation, non-coding RNAs, and post-translational modifications (PTMs). Histone proteins are small, approximately 14.5-kb proteins with an N-terminus “histone tail” formed from charged lysine (K) and arginine (A) amino groups that form sites for PTMs.

### How DNA Methyltransferases Interact with Histone Modification Proteins to Regulate Gene Expression

The generation of heritable marks on DNA and histone tails is an epigenetic regulatory process fundamental to stable gene expression; alterations in these marks can, therefore, have deleterious effects. The nitrogenous bases cytosine (C) and guanine (G) play crucial roles in DNA regulation; they are commonly situated in tandem as sequential GCGCGCGCG repeats known as CpG islands, which act as regulatory hotspots found upstream of gene promoter regions. Three DNA methyltransferases (DNMTs) control methyl group transfer or CpG island methylation: DNMT1, which is generally associated with the methylation of imprinted parental genes, and DNMT3 A/B, which are responsible for methylating DNA to regulate gene expression. In contrast to DNA methylation, which is associated with gene silencing, histone methylation, acetylation, and phosphorylation can both inhibit (silence) and promote (activate) transcription through histone N-terminal tails, which are rich in arginine (A) and lysine (K) amino acids. The histone tail serves as a hotspot for PTMs such as methylation in chromatin-associated transcriptional regulation [22], not least because lysines can be mono-, di-, and tri-methylated.

Histone methyltransferases (HMTs) and histone deacetylases work closely to regulate the gene silencing. In eukaryotic transcription, genes can be switched on or off in several different ways. SETDB1 and DNMTs reciprocally interact with each other through a variety of signaling pathways at CpG dinucleotide regions [23]. DNMTs silence genes by recruiting methyl-CpG-binding domain proteins (MBDs) to CpG islands for methyl transfer. An MBD-containing protein is then signaled and sequestered to the DNMT CpG region. For example, HMTs such as SETDB1 contain an N-terminal MBD region and are signaled to the DNMT CpG site for binding and activation. The activation of the MBD–SETDB1 complex signals methyltransferase activity by recruiting the co-factors *S*-adenosylhomomethionine (SAM) and homolog of murine ATFa (Activating Transcription Factor a)-associated modulator (hAM) (see below) to methylate the lysine tail of the histone protein. An S_N_2 (substitution nucleophilic 2) reaction then causes conformational changes in both the HMT and the lysine residue, which becomes positive, as seen in H4K9me, H3K9me3, and H3K27me3. Moreover, this chemical modification of euchromatin usually activates heterochromatin protein-1 (HP-1), which induces heterochromatization and effectively silences these loci by condensing the histone protein [23]. An example of the SETDB1–DNMT relationship can be seen in work by Li et al. who highlighted the role of SETDB1 and DNMTs in HeLa and MDA-MB-231 cancer cell lines. It was shown that SETDB1 and DNMT3a directly interact and localize at the promoter regions of tumor suppressors and induce heterochromatin formation and gene silencing [24]. Specifically, this interaction was identified to occur at the N-terminus of SETDB1 and the plant homeodomain of DNMT3a. Furthermore, it was observed that both SETDB1 and DNMT3a expression is essential for gene repression and that both SETDB1 and DNMT3a occupy the P53BP2 (p53 binding protein 2) and RASSF1A (Ras-associated domain family 1A) tumor suppressor loci, thus demonstrating the direct interaction of both SETDB1 and DNMT3a to functionally play a role in endogenous DNA CpG dinucleotide methylation and histone lysine trimethylation [24]. This highlights the effective dynamics of DNMT3a and SETDB1 in playing a pivotal role in the regulation of genes, as well as gene silencing.

## 3. SETDB1: A Nuclear Transcriptional Regulator

### 3.1. Structural and Functional Biochemistry of SETDB1 

**Figure 1:**
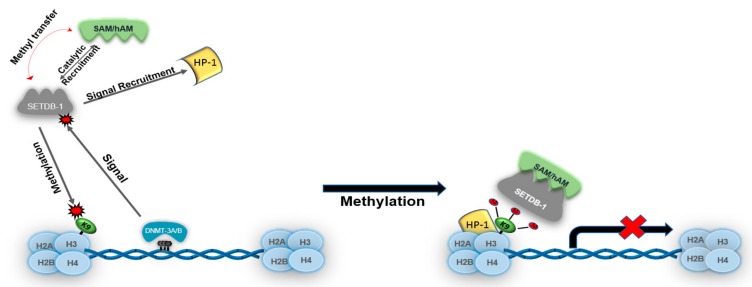
DNA-methyltransferase 3 A/B (DMNT-3 A/B) CpG island-mediated signaling of SET Domain Bifurcated Histone Lysine Methyltransferase 1 (SETDB1). SETDB1 methyltransferase activity is activated through signaling via DNMT-3 A/B. SETDB1 catalytically recruits *S*-adenosyl-homocysteine (SAH) and homolog of murine ATFa (Activating Transcription Factor a)-associated modulator (hAM) as methyl donors. Histone 3 lysine residue 9 (H3K9me) is then di- and tri-methylated, which leads to euchromatin being condensed into heterochromatin to repress transcription.

SET (Su(var)3-9, Enhancer of Zeste, Trithorax) domain bifurcated histone lysine methyltransferase 1 (SETDB1), also known as Erg-associated SET domain (ESET), was first discovered by Yang et al. in hybridized yeast showing Erg, an erythroblast transformation-specific (ETS) transcription factor superfamily member, associating with the SET domain protein via N-terminal interactions [18]. SETDB1 maps to chromosome 1q21, is 1291 amino acids in length, has a molecular weight of 143.1 kD, and is 39 kb in humans and 36 kb in mice. Mouse ESET shares 92% similarity with human SETDB1. Human SETDB1 is responsible for the di- and tri-methylation of H3K9 via the co-factors SAM and hAM (Figure 1). These cofactors exert catalytic activity in the C-terminus of the SET domain. Upon activation, SAM and hAM bind to the substrate-binding site of SET and transfer methyl (–CH_3_) groups via an S_N_2 reaction to the SET domain protein, which then methylates residues on the histone protein [25]. This activates HP-1, which binds to the histone to induce heterochromatization and form various protein complexes that repress gene expression.

**Figure 2:**
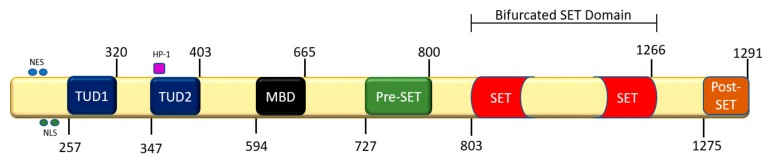
Schematic of SETDB1 domains inclusive of amino-acid location. Blue circles = Nuclear Export Sequence (NES), green circles = NLS (nuclear recognition sequences), purple square = heterochromatin protein-1 (HP-1).

As with other HMTs, SETDB1 contains an evolutionarily conserved SET domain specific to that enzyme. However, the C-terminus of SETDB1 contains pre-SET, SET, and post-SET domains that are responsible for its catalytic activity. Uniquely, SETDB1 has a bifurcated SET domain interrupted by a sequence several hundred amino acids long that is evolutionarily conserved in SETDB1 (Figure 2) [18]. The function of the interrupting sequence is yet to be established, but mutations in the C-terminus of SETDB1 were reported to diminish its methyltransferase activity. However, C-terminus mutations do not drastically affect methylation patterns, suggesting that there might be redundancy with other HMTs. Furthermore, the N-terminus of SETDB1 contains a methyl-CpG-binding domain (MBD) and two consecutive Tudor domains, the MBD domain being of particular interest as it interacts with DNMT and may be responsible for the intramolecular coupling of CpG dinucleotide sites on complementary DNA strands. This binding sequesters HMTs to euchromatic regions to methylate histone proteins, resulting in heterochromatization via orientation of histone protein 1 and the formation of other complexes (HP-1). Conversely, the SETDB1 Tudor domains are responsible for anchoring SETDB1 to lysine and arginine sites, RNA metabolism, and germ cell development [26]. Tudor domains also play a significant role in SETDB1’s interaction with other regulatory complexes such as the histone deacetylases (HDACs) 1/2 and the co-repressors mSin39h1 and KRAB-ZPF–KAP-1 (Kruppel-Associated Box-Zinc finger proteins-KRAB-associated protei Dodge, J.E.; Kang, Y.-K.; Beppu, H.; Lei, H.; Li, E.n-1), which form large multi-protein complexes that regulate gene expression at promoter sequences of euchromatic genes [27].

SETDB1 acts as a transcriptional regulator; however, its own promoter expression and transcription also stimulated interest. The promoter region of SETDB1 sits 118 bp upstream from an ATG start codon and is terminated via a TAG stop codon. Interestingly, the promoter sequence of SETDB1, unlike traditional genes, lacks TATA and CAAT, which act as promoter sequences for transcription factors to bind and signal RNA polymerase II in eukaryotic cells [28,29]. The SETDB1 promoter region is GC-rich, which provides binding sites for the transcription factors GATA binding protein-1 (GATA-1), nuclear factor Y (NF-Y), and specificity protein 1 (SP-1). SETDB1 is, therefore, likely to have a housekeeping function. Interestingly, the three SETDB1 splice variants also exert a multitude of biological features similar to the full-length product, although the full scope of their action is currently unknown. Isoform 1, also known as full-length SETDB1, contains all the necessary exons and N- and C-terminus domains, while isoform 3 lacks the C-terminus responsible for catalysis [30]. Using isoform expression analysis in immortalized mouse cell lines (TM3, TM4, NIH3T3, 3T3-L1, LG, and p53^−/−^), the relative expression of SETDB1 isoforms was measured and shown to exhibit oscillating expression relative to normal and cancer cell lines [30]. Splice variant isoform 3 was more abundant in the immortalized cell lines than full-length isoform 1, and isoform 1 was more abundant in normal tissues such as the testis and liver. However, compared to relative isoform expression in normal and immortalized cell lines, full-length SETDB1, similar to PR (PRDI-BF1 and RIZ1 homology) domain-containing HMTs, may be tumor-suppressive, whereas the splice variant was overexpressed in the immortalized cell lines [30].

### 3.2. Localization of SETDB1

SETDB1 also shows variable localization in cultured cells. Schultz et al. demonstrated that SETDB1 has diffuse nuclear expression, consistent with its action as a transcriptional regulator [27]. Cho et al. and Yeap et al. demonstrated that SETDB1 can also display a punctate nuclear pattern [31,32]. These patterns are not mutually exclusive. The exact cause and effect of the punctate pattern is yet to be determined, but may be due to the interactions of promyelocytic leukemia nuclear bodies (PML-NBs), which play multiple roles in genome maintenance [31]. Moreover, Loyola et al. established that this diffuse pattern is likely to be due to SETDB1 localizing to the pericentric area of heterochromatin in a cell-cycle-dependent manner [33], suggesting that SETDB1 as a transcriptional regulator via promoter regulation has other functions [34]. Furthermore, Blackburn et al. showed that differential SETDB1 localization may be due to the existence of splice variants with different nuclear localisation sequences (NLS) and nuclear export sequences (NES) [30].

Intracellular localization is tightly regulated by nuclear-to-cytoplasmic shuttling. Protein shuttling plays an important role in maintaining normal cellular function and is carried out via a multitude of nuclear export proteins including exportin-1 (EXPO-1), also known as chromosomal region maintenance 1 (crm-1), which mediates protein transfer through nuclear pore complexes (NPCs) [35]. To further understand the nuclear localization of SETDB1, Cho et al. monitored endogenous SETDB1 levels relative to nuclear location. Unexpectedly, overexpressed SETDB1 was shunted into the cytoplasm [31,36], and treatment with a nuclear export protein inhibitor leptomycin B (a crm-1-specific inhibitor) was used to determine that GFP-bound SETDB1 entry via NPCs resulted in a punctate localization at PML-NB foci, as well as a diffuse pattern in the nucleoplasm [31,32]. However, excess GFP–SETDB1 was exported in a crm-1-specific manner, illustrating that exogenous SETDB1 nuclear entry is tightly regulated.

As reviewed in Kang et al., the existence of NLS and NES domains in SETDB1 illustrates that nuclear–cytoplasmic shuttling of SETDB1 is possible [34]. Deletion of the NLS and NES in the N-terminal amino region of SETDB1 diminishes the cytoplasmic retention of SETDB1, resulting in the abundant production of nuclear and cytoplasmic SETDB1. Furthermore, cell fractionation experiments highlighted the variation in localization of SETDB1 and the other histone methyltransferases containing SET domains (G9a/GLP (G9a-like protein) and Suv38h1). Endogenous SETDB1 seems to have both cytoplasmic and nuclear functions, perhaps by interacting with cytoplasmic intracellular signaling pathways such as TGF-β. This functionality demonstrates that the uniqueness of SETDB1 is reserved for its nuclear activity [34].

### 3.3. The Nuclear Role of SETDB1

SETDB1 has diverse nuclear roles. However, SETDB1 seems to rely heavily on its interacting partners for its regulatory effect. Furthermore, ablation of the interacting complex junctions between SETDB1 and its neighboring regulators can result in epigenomic dysregulation.

#### 3.3.1. SETDB1 Is Protected against Proteasomal Degradation by ATF71P

The various complexes formed by SETDB1 were thoroughly investigated. Although the underlying mechanisms that facilitate gene silencing are not fully understood, SETDB1 is thought to be a critical component of many genomic regulators. The human silencing hub, HUSH, is a SETDB1-interacting complex involved in transgene silencing. SETDB1 is recruited by HUSH complexes at heterochromatic loci to tri-methylate H3K9. The activating transcription factor 7-interacting protein (ATF71P), also known as MCAF-1 (Methyl-CpG-Binding Domain Protein 1 (MBD1)-containing chromatin associated factor 1) or mAM/hAM, is a prominent co-factor for the tri-methylation of H3K9me [37]. However, other factors may be involved in trimethylation, and ATF7IP may play a protective role against proteasomal degradation of SETDB1 [38]. Recent investigations into the localization and interactions of SETDB1 show that ATF71P binds to SETDB1 in the nucleus, thereby preventing proteasomal degradation, as the loss of ATF71P results in cytoplasmic accumulation of SETDB1. Furthermore, the relative abundances of SETDB1 and ATF71P are directly proportional, indicating that the nuclear stability of SETDB1 and ATF71P shows a degree of mutual exclusivity [39].

#### 3.3.2. Function of SETDB1 Interactions with Promyelocytic Leukemia Nuclear Bodies (PML-NBs) 

As discussed above, SETDB1 is a H3K9 methyltransferase that associates with various transcriptional regulators, most notably Kap-1, HDAC 1/2, mSin3 A/B, and DNMT-3 [40]. These transcription factors direct SETDB1 to pericentric heterochromatic regions to silence genes and regulate gene expression. This silencing function of SETDB1 shows an intricate relationship with the activation of HP-1, which binds H3 to permit heterochromatin formation and in turn gene repression.

SETDB1 also plays an intricate role in early embryonic development (see below) and the structure and regulation of PML-NBs [36]. PML-NBs are matrix-associated domains roughly 0.2–1.0 nm wide that play a vital role in maintaining cellular and genomic stability by directly interacting with downstream pathways including transcription, pro-viral silencing, apoptosis, senescence, angiogenesis, and tumor suppression [41]. Rigorous studies on SETDB1–PML-NB stability established that SETDB1 is a key mediator of the structural integrity of PML-NBs in many biological processes. Ablation of either SETDB1 or PML-NBs leads to the reciprocal dismantling of each protein [36,42], and immunoprecipitation experiments showed that SETDB1 and PML physically interact [36]. Therefore, SETDB1 is a critical protein stabilizer in many signaling pathways.

#### 3.3.3. SETDB1 Has the Ability to Regulate Viral Transcription

Histone tail modifications orchestrate chromatin composition as part of cellular homeostasis. Many pathogens manipulate host regulatory mechanisms for survival. Viral integration into the host genome can contribute to carcinogenesis and cellular dysregulation. For example, the human immunodeficiency virus (HIV) is a 110-nm encapsulated lentivirus that causes immune dysregulation and anergy. The HIV genome consists of nine viral genes that integrate into coding DNA. HIV-1 utilizes the host cellular machinery to transcribe its own genes [43]. The HIV-1 coding region is expressed by a single promoter located within the viral long terminal repeat (LTR) [44]. Binding and regulation of this promoter region is under the influence of the trans-activator *TAT*, with the Tat protein promoting replication of the viral genome by binding to the LTR of viral messenger RNA (mRNA), also known as the transcriptional activation region (TAR). Tat–TAR binding releases transcriptional repression to increase viral RNA transcription. The Tat protein can undergo various PTMs that ultimately affect the rate of transcription, making it a potential therapeutic target. Recently, van Duyne et al. demonstrated that Tat can undergo methylation at lysine residues 50 and 51 through SETDB1 methylation, which corresponds to a decrease in viral replication by inhibiting promoter activity.

## 4. Epigenomic Functions and Interactions of SETDB1

SETDB1 is a prominent di- and tri-methylator of H3K9, which is important for the control and regulation of euchromatic promoter regions throughout the genome for gene silencing [45]. SETDB1 participates in many processes involved in embryonic development and genome maintenance, with studies of early embryogenesis, X inactivation, and proliferation/differentiation control emphasizing SETDB1’s role as a critical epigenomic protein in normal physiology [46].

In addition to direct chromatin binding, SETDB1 directly interacts with pro-repressive proteins and complexes within the promoter regions of euchromatic DNA [47]. These interactions initiate the recruitment of heterochromatin-associated proteins that induce gene silencing [48], although the exact mechanism via which SETDB1 directly binds DNA is yet to be fully established.

### 4.1. SETDB1 Is Associated with Transcriptional Modulators 

**Figure 3:**
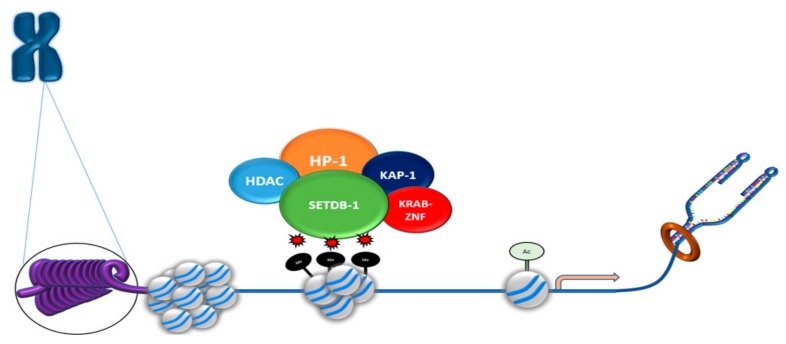
SETDB1 interacts with transcriptional regulators to induce heterochromatin formation. SETDB1 tri-methylation of histone tails is a multifactorial process that requires the orchestrated recruitment and interactions of various chromatin proteins to repress transcription.

Various transcriptional regulators interact across the genome to ensure tightly controlled transcription and genome integrity. SETDB1 interacts with an important transcriptional complex, KRAB-ZFP–KAP-1, to orchestrate genetic control (Figure 3). KRAB-ZPF plays various roles in transcriptional regulation. It was shown to be a vital moderator of early transposable element (TE) regulation through recruitment of KAP-1 (Tripartite Motif Containing 28 (TRIM28)), as well as signaling SETDB1 binding [49]. An important characteristic of KRAB-ZPF in early development is its ability to target specific DNA loci and induce KAP-1-mediated heterochromatin formation [50]. KAP-1 recruitment is a vital component in the regulation of TE expression [50]. Loss of KAP-1 and its conjugate HMT SETDB1 has deleterious effects that lead to uncontrolled TE expression in embryonic stem cells (ESCs), which then disrupts maturation [51]. SETDB1–KAP-1 interactions are clearly important in the activation of transcriptional silencing.

SETDB1, via its overlapping N-terminal Tudor domains [40], also associates with other transcriptional regulators including histone deacetylases (HDAC1 and HDAC2) and the transcriptional co-repressor mSin3A/B, which form large protein complexes that play pivotal roles in transcriptional silencing.

### 4.2. SETDB1 Interacts with Early Embryological Machinery

In mammals, several H3K9 methyltransferases are responsible for suppressing transcription and regulating differentiation pathways in developing cells via protein–protein interactions. In mouse models, SETDB1 was shown to be a crucial player in early development. Dodge et al. demonstrated that homozygous knockout of SETDB1 during the pre-implantation stage was lethal [52]. During oogenesis, SETDB1 is maternally expressed at high levels in the embryo and aids in its development. Moreover, Cho et al. demonstrated that embryonic SETDB1 is not actively transcribed until the blastocyst stage [31]. SETDB1 is essential for maintaining pluripotency and repressing trophectodermal differentiation in ESCs and for primordial germ cell and neuronal progenitor development. In addition, SETDB1 double knockouts highlighted the role of H3K9me3 as a critical meiotic and cell-cycle regulator in mouse oocytes and embryos; SETDB1 knockdown impairs meiosis by delaying meiotic resumption via defects in protein kinase A (PKA) activation and maturation, promoting factor re-establishment after phosphorylation-mediated silencing. Furthermore, chromosomal condensation and kinetochore–spindle interactions were also impacted by SETDB1 knockdown, affecting not only segregation dynamics but also the cell cycle.

Finally, SETDB1’s H3K9me3 functionality was shown to be responsible for silencing class 1, 2, and class 3 endogenous retrotransposon sites throughout the genome. Karimi et al. and Tan et al. demonstrated that SETDB1 during early development suppresses endogenous retroviral LTRs by silencing endogenous retroviral element (ERV)-driven expression of nearby genes [53,54]. Furthermore, Eymery et al. demonstrated that SETDB1 is a key mediator of the downregulation (but not silencing) of class 1 and class 3 retrotransposons (ERVK, ERVL-MaLR) (Endogenous retrovirus-K promoter, endogenous retrovirus related elements and mammalian apparent LTR-retrotransposons) during oogenesis [55]. SETDB1 is a key component of endogenous pro-viral silencing and genomic stability during early development and embryogenesis.

### 4.3. SETDB1 Dictates T-Cell Development and Linage Commitment 

Until recently, the impact of histone modifications on immune system development and physiology was poorly understood. Recently, however, Takikita et al. and Adoue et al. demonstrated that SETDB1 H3K9 methylation impacts thymocyte maturation and influences T-helper cell lineage specification [56,57].

Conventionally, histone protein methylation by members of the Suv39h1 methyltransferase family was shown to be dispensable with respect to T-cell differentiation and maturation [56]. Nevertheless, Takikita et al. demonstrated that SETDB1 influences T-cell development by regulating various intracellular signaling genes [57]. The ERK (extracellular-signal-regulated kinase) pathway is involved in the positive selection of CD4 and CD8 T cells. Knockout SETDB1 in thymocytes abolished ERK signaling by upregulating the natural inhibitor Fcγ receptor IIB (FcγRIIB), following which apoptosis of single-positive CD4+ and CD8+ T cells was observed. Upon FcγRIIB depletion in SETDB1−/− cells, ERK signaling was rescued and selection was moderately stabilized, indicating that SETDB1 critically influences positive selection and potentially plays a role in immunological tolerance [57]. Furthermore, the epigenetic events occurring in naïve CD4+ selection were also investigated. Adoue et al. illustrated that SETDB1-mediated methylation of H3K9me3 heavily impacted the promotion and initiation of lineage commitment [56]. Upon encountering antigens and the major histocompatibility complex (MHC) through interactions with the T cell receptor (TCR), naïve CD4+ T cells undergo differentiation into T-helper cell subsets (Th1, Th17, Th2, Tfh, and T-regulatory cells). This cognate interaction activates SETDB1 signaling and tri-methylation of ERVs flanking Th1 enhancer regions, inhibiting the transcription of the transcription factor T-bet and promoting Th2 helper lineage commitment. Thus, SETDB1 H3K9me3 activity is crucial in early maturation, positive selection, and lineage integrity of T cells [56,57]. This implicates SETDB1 as a potential target for cancer vaccines, as inducing CD4+ T cells could support the expansion of CD8+ T cells in the tumor [58].

## 5. Structural and Functional Dysregulation of SETDB1 Due to Mutations 

Schultz et al. demonstrated the existence of three C-terminal *SETDB1* mutants, H1224K, C1226A, and C1279Y, which impair SETDB1 H3K9 methyltransferase activity through single amino-acid substitutions at highly conserved residues within the catalytic domain of SETDB1 (Table 1) [27]. H1224K and C1226A mutations were reported in accelerated melanoma in zebrafish [59]. These mutants show that, although catalytically inactive, mutant SETDB1 can still bind to G9a/GLP and SUV39h1 to carry out its methylation functions. In the absence of SETDB1’s catalytic capabilities, other HTMs appear to be able to methylate target genes and/or molecules [27].

Kang et al. recently performed whole-genome sequencing of 69 patient malignant pleural mesotheliomas (MPMs) and identified seven somatic mutations in SETDB1 across the entire protein [60]: four point mutations and three deletions. Of these deletions, two shared the same N-terminal 17 bp deletion (P226RfsX4) and one in-frame C-terminus post-SET domain deletion (F1250del). The three missense mutations were located in the C-terminus region and, interestingly, within the bifurcated SET domain (G869E, C911F, and S947C) with known pathophysiological functions. In addition, an in-frame deletion resulting in truncation of SETDB1 (K674SfsX73) and a non-sense premature stop codon mutation were also found (Y249X).

## 6. Epigenetic Influence of SETDB1 in Tumorigenesis

In addition to mutations, epigenetic events such as PTMs heavily impact the tumorigenic phenotype [15]. SETDB1 is implicated as an oncogene due to its implicit role in regulating gene silencing via H3K9 methylation and influencing chromatin factors that regulate tumorigenesis.

### 6.1. SETDB1 Is Associated with Cancer Pathway Activation 

Protein kinases (PKs) are responsible for the phosphorylation of second messenger proteins that promote intracellular signaling of pathways implicated in survival, proliferation, metabolism, and intracellular communication. Protein kinase B (AKT) is a serine/threonine-specific protein kinase that is part of the PI3K (Phosphoinositide 3-kinase) /AKT/mTor (mammalian target of rapamycin) pathway, a crucial cell signaling pathway that, when abnormally activated, can be oncogenic [61]. SETDB1 plays a critical role in the activation, cell membrane recruitment, and phosphorylation of AKT by directly methylating its lysine residues. AKT is activated in response to growth factors acting upon intramembranous tyrosine kinase receptors that, upon stimulation, phosphorylate intracellular tyrosine kinase residues which then propagate to downstream signaling proteins. AKT interacts with various other signaling pathways, most notably MAPK (mitogen-activated protein kinases) at the TKR (Tyrosine Lysine Arginine) residue via TKR phosphorylation, leading to RAS-GTP (rat sarcoma- guanosine triphosphate) recruitment (through adapter proteins Grb-2 and SOS (son of sevenless)). However, the mechanisms behind the activation and translocation of AKT are poorly understood. Wang et al. recently proposed a model of cytoplasmic AKT activation [62], in which the early activation of AKT is critically dependent on SETDB1’s methylation functions and its counteracting demethylase JMJD2A. SETDB1 methylates inactive AKT at K64 in response to growth factor stimulation, which serves as a scaffold for the recruitment of the JMJD2A adapter. Upon AKT-mediated K64 methylation, JMJD2A then binds to SETDB1/AKT, forming a complex, which then recruits the vital ubiquitin E3 ligase enzymes tumor-necrosis factor receptor-associated factor 6 (TRAF6) and S-phase kinase-associated protein-2 - Skp1-Cullin-1-F-Box (Skp2-SCF) to bind to AKT. Following ubiquitination via E3 ligases, AKT is partially activated via cytosol to membrane translocation [63]. SETDB1-mediated AKT activation might, therefore, contribute to AKT’s oncogenic activity.

Furthermore, Guo et al. demonstrated that SETDB1 was a critical regulator of AKT by synergizing with PI3K in the early upstream part of the pathway [64]. SETDB1-mediated methylation of K140 and K142 enhanced AKT1 activation and sustained its protein kinase activity. Additionally, SETDB1 also influenced AKT translocation by enhancing AKT–TRAF-6 activity. SETDB1 and AKT interactions were found to be mediated by SETDB1’s N-terminal Tudor domains and linker regions of AKT1, which stabilized the AKT1–SETDB1 complex. However, disproportionate increases in SETDB1 activity reciprocally increased AKT1 methylation and, in turn, increased AKT1 activity. AKT1 hyperactivation is implicated in many cancers and generally indicates an unfavorable prognosis [62]. Furthermore, Guo et al. demonstrated that JMJD2 demethylase plays a vital role in maintaining AKT signaling. The demethylase JMJD2 also known as KDM4B was capable of demethylating AKT, which significantly reduced its signaling, as well as partly restoring cellular homeostasis, with KDM4B antagonizing SETDB1’s methylation properties. AKT is a bona fide methylation and demethylation non-histone substrate for the SETDB1–KDM4B axis [64].

### 6.2. Epigenetic Influence of SETDB1 in Breast Cancer 

SETDB1 and other HMTs play an important role in gene expression and transcription via heterochromatin and euchromatin alterations through post-translational modifications of histone tails [65]. This makes them an attractive target for breast cancer therapy, since epigenetic aberrations are implicated in the development and progression of breast cancer metastasis. As well as influencing breast cancer cell migration, invasion, and proliferation [66], SETDB1 affects stem-cell metabolism/lineage, thus influencing metastatic development.

**Figure 4:**
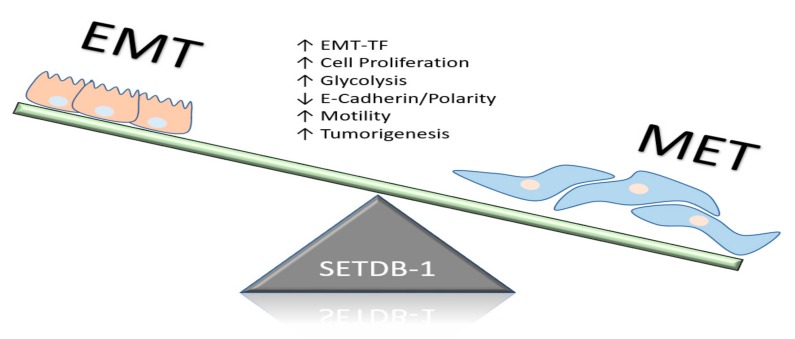
SETDB1’s influence on epithelial-to-mesenchymal transition (EMT) programs that initiate metastasis. This metabolic reprogramming altered the breast cancer phenotype and contributed to progression through acquisition of cancer stem cell characteristics.

Recently, Ryu et al. demonstrated that the expression of SETDB1 was responsible for the regulation of metastatic marker attainment in breast cancer [19]. SETDB1 was shown to indirectly regulate TGF-β levels through SMAD-7, a key regulatory signaling gene [19]. The TGF-β signaling network is a signal transduction pathway known for host immune regulation, promotion of EMT, and other functions [67]. The SETDB1–SMAD-7 interaction was shown to influence cell proliferation, migration, and invasion of breast cancer cells through the activation of EMT programs (Figure 4). In MBA-MB-231 cell lines, SETDB1 promoted EMT programs by inhibiting the epithelial markers E-cadherin and claudin-1 and upregulating the mesenchymal markers N-cadherin and vimentin. Furthermore, Wu et al. confirmed in the MCF-7 and MDA-MB-231 human breast cancer cell lines that SETDB1 amplification was associated with metastatic progression [68]. SETDB1 appears to be a central regulator of breast cancer metastasis through the acquisition of stem-cell-like properties, as well as manipulating EMT programs. The relative expression of SETDB1 in mouse NMuMG mammary epithelial cells directly repressed EMT programs through the recruitment of SMAD-3 in a TGF-β-dependent manner, which in turn regulated the activity of EMT transcription factors. The SETDB1/SMAD-3 complex interaction repressed the key EMT promoter complex SMAD3/4 and inhibited the expression of EMT-TF Snail through H3K9 methylation. Thus, SETDB1 expression is important for the maintenance of EMT programming and the acquisition of stem-cell-like properties that facilitate metastasis [69].

### 6.3. SETDB1 Methyltransferase Activity Sustains Hematopoietic and Progenitor Stem Cell Lineages 

In mammals, the continued maintenance of the genome and its biological integrity is sustained through crosstalk between related PTMs on DNA and histones. This evolutionarily developed molecular signaling network is crucial to stem-cell development and differentiation. The development of hematopoietic stem cells (HSCs) and progenitor cells is largely impacted by their environment. While their pluripotency provides cellular diversity, a key characteristic of stem cells, it is also implicated in cancer dissemination [70].

The development of lineage specificity is highly dependent on molecular signaling interactions including nuclear PTMs. Recently, Miyagi et al. demonstrated that the epigenetic influence of the transcriptome and epigenome scaffold protein KAP-1 (TRIM-28) is essential for the maintenance of HSC development. KAP-1 has diverse roles including interacting with KRAB-ZNF motifs which, in turn, recruit SETDB1 to repress ERVs [71] within euchromatic regions and serve to maintain heterochromatic regions. Throughout HSC differentiation, euchromatin and heterochromatin levels oscillate, although heterochromatin is more abundant than euchromatin during HSC differentiation [72].

G9a and SETDB1 are a part of the same family of histone methyltransferases, SUV39 [73]. Recently, G9a inhibition prevented heterochromatin formation in HSCs and delayed differentiation [74], demonstrating that H3K9 methylation impacts euchromatin and heterochromatin levels in HSCs and may influence differentiation. Furthermore, SETDB1 was shown to be highly active in HSC maintenance [75] and, similar to KAP-1, SETDB1 plays a pivotal role in maintaining HSCs and progenitor cells during differentiation by inhibiting nonhematopoietic metabolic genes, for example, fructose-1,6-bisphosphate 1 (Fbp1) and Fbp2, in an SETDB1–KAP-1–HP-1-dependent manner. SETDB1 appears to play a fundamental role in maintaining HSCs in a metabolism-specific manner [75].

### 6.4. The Influence of SETDB1 on Stem and Cancer Cell Metabolism and Its Consequence on the Warburg Effect 

ATP can be generated via several metabolic processes such as glycolysis, fatty-acid oxidation, the citric acid cycle, and the pentose phosphate pathway [76]. SETDB1 maintains glycolysis by depositing H3K9me3 signatures on *fbp1/2* genes, thus inhibiting gluconeogenesis and progressing through glycolysis by the enzyme phosphofructokinase [75].

Metabolic reprogramming in cancer was first described by Warburg [77]. Dong et al. described that epigenetic reprogramming by the key HMT G9a interacted with Snail and DNMT to form a repressor complex that resulted in loss of FBP-1 in breast cancer cells [78].

## 7. SETDB1 as a Therapeutic Target

The epigenome is a particularly attractive therapeutic target because epigenetic alterations are reversible. Current proprietary inhibitors and conventional chemotherapeutics exhibit a variety of off-target toxic effects and resistance [79]. However, epigenetic inhibitors may offer some advantages over traditional chemotherapies, particularly if they target the stem-cell compartment responsible for migration, becoming quiescent, and conferring resistance [80] either immediately or after late re-activation [81].

In particular, histone lysine methylases are highly attractive therapeutic targets as small-molecule inhibitors due to the specificity of the targets. The main histone lysine methylase inhibitors currently approved by the United States (US) Food and Drug Administration (FDA) targets enhancer of zeste 2 polycomb repressive complex 2 subunit (EZH2), G9a/GLP, disruptor of telomeric silencing 1-like (DOT-1L), and SUV39h1 [82]. At present, there is no specific inhibitor for SETDB1. Studies use non-specific inhibitors such as 3’-deazaneplanocin A (DZNep), mithramycin A, paclitaxel, and microRNA miR-381-3p, although these show a variety of off-target effects and resistance can occur [83,84,85]. In a breast cancer study, the microRNA miR-381-3p successfully downregulated SETDB1 expression, but once SETDB1 expression was restored, SETDB1 overcame miRNA interference, indicating SETDB1 as a potential target for breast cancer therapy [68].

G9a, also known as EHMT2, is an H3K9me3 part of the SUVh391 family of histone methyltransferases. Recent clinical work surrounding G9a and its heterodimer GLP (G9a-like protein) showed it to have a significant impact in the cancer landscape. Recently, Ho et al. demonstrated the use of the G9a-specific inhibitor BIX-01294 to effectively abrogate G9a’s actions in the breast cancer model [86]. This in turn promoted apoptosis and impaired cell migration, cell cycle, and anchorage-dependent growth in breast cancer cells. However, unexpectedly, G9a inhibition also led to the induction of pro-tumorigenic pathways such as hypoxia-induced pathway (HIF), even in normoxic conditions [86]. Although G9a may be effective in managing tumor cell growth, the activation of the HIF pathway is closely correlated to increased cell survival and increased metastasis and cancer aggressiveness [87], thus reducing the therapeutic effect of its specific inhibitor. This, therefore, signifies the importance for further investigating in histone methyltransferases, such as SETDB1, in breast cancer development. Furthermore, currently, there are no histone methyltransferases in clinical trials for the treatment of breast cancer, further highlighting the need for further investigation for histone methyltransferase-associated breast cancers, as well as the development of therapeutics targeting these enzymes.

The therapeutic intervention of epigenetic regulators is currently of heavy interest; however, the specificity for drug target binding remains a challenge. DZNep is an *S*’-adenosyl methionine inhibitor (sAM) that is pharmacodynamically diverse. In the human genome, DZNep works by altering the genomic and epigenetic landscape. It is documented to be an effective H3K27me3 inhibitor, most notably EZH2, and H3K9me3 [83]. However, its actions are limited and it is ineffective in other HMTases such as H3K4me3 [83]. Furthermore, the diverse biological nature of DZNep led to cumulating interest in its ability to effectively reverse carcinogenesis through the induction of apoptotic pathways in cancer cells via accumulation of reactive oxygen species (ROS) and actions on apoptosis-associated genes (F-Box Protein 32 (FBXO32), p16, p31, and p27). DZNep is a well-known inhibitor of the H3K27me3 histone methyltransferase EZH2 but its treatment also negatively regulates SETDB1 by downregulating its expression in various cancer cells lines, a mechanism that is also employed by the inhibitor mithramycin A and paclitaxel [66,83,85]. Recently, Lee et al. investigated the efficacy of DZNep on SETDB1 in human cancer cell lines. It was seen that DZNep induced growth inhibition and increased apoptotic events in the cell lines and reciprocally decreased SETDB-1 expression by binding to the promoter region of the SETDB1 gene, thus inhibiting its expression. This highlighted that DZNep, as a non-specific inhibitor, is capable of targeting multiple HMTases and decreasing expression levels, as well as inducing apoptosis in lung cancer cells. However, its specificity is a limiting factor [88,89,90].

Paclitaxel, a taxane, is a well-known chemotherapeutic drug known for its role in the GTP-independent tubulin stability and cell arrest capabilities [91]. A question around paclitaxel is its effect on chromatin stability. Recently, Noh et al. discovered that the introduction of paclitaxel into human cancer cells led to the reduction of H3K9me3 marks via SETDB1 transcriptional downregulation, as well as induction of p53 leading to cell death in a G2/M arrest manner. This signified paclitaxel’s ability to activate p53 expression, as well as preferentially bind to the promoter region of SETDB1 and downregulate its expression [85]. Nevertheless, the characteristic interactions between paclitaxel and the regulatory events that occur in breast cancer are not well characterized.

The guanosine-cytosine-rich DNA-binding antineoplastic drug, mithramycin A, is a selective specificity protein (Sp) inhibitor. It is a part of a family of zinc finger transcription factors that are known to regulate transcription of several tumorigenesis-related genes [92]. It is a transcription factor that binds to GC-rich promoter regions of oncogenic genes, such as SETDB1. Similarly to Sp-1, mithramycin mimics its binding actions and competes for GC-rich binding sites. This in turn downregulates Sp-1-related genes. Recently, the study of mithramycin in various cell lines demonstrated it to have radical effects on SETDB1 expression, as well as H3K9me3 signatures [84]. For example, the SETDB-1–AKT axis plays a critical role in cell regulation dynamics, as well as tumorigenesis. Following mithramycin implementation, the methylation and phosphorylation signals were reduced, thus reducing oncogenic AKT signaling and interactions with PDK-1. Furthermore, it was also shown that, through xenograft mouse experiments, the mice given mithramycin analogs had significantly reduced tumor sizes [64]. However, although the bulk tumor levels may have been reduced, this phenomenon alone does not warrant it to be sufficient evidence in categorizing mithramycin A as specific SETDB1 target or in suggesting it to abrogate CSC development. Recently, a phase II clinical trial demonstrated the use of the AKT inhibitor MK-2066 in patients with advanced breast cancer who have AKT/P13K and phosphatase and tensin homolog (PTEN) mutations. At present, evidence suggests patients to have a partial response to the inhibition of AKT mutations and to have limited clinical activity in advanced breast cancer due to a variety of situations surrounding tumor heterogeneity [93]. This, therefore, highlights the potential use for combination therapy of AKT inhibitors, as well as histone methyltransferases such as SETDB1, to potentially investigate the heterogenic events occurring in cancer development. As previously mentioned, SETDB1 interacts with the AKT pathway and, therefore, may be a relevant point for investigation.

Undoubtedly, there is huge potential in developing small-molecule inhibitors against histone methyltransferases, and a deeper understanding of the exact mechanism of inhibition will allow us to develop therapeutics that are highly targeted and effective. Needless to say, SETDB1 would work in conjunction with other factors to regulate gene expression. Specifically, given the SETDB1–AKT axis (discussed in Section 6.1), there is great interest in examining the cooperation between protein kinases and histone methylases to mediate expression of oncogenes and immune genes to counteract the effects of cancer cells. In particular, our lab showed that not only does protein kinase C-theta (PKC-θ) control and regulate Lysine-specific histone demethylase 1 (LSD1) phosphorylation, but LSD1 itself couples to PKC-θ to promote LSD1-mediated gene expression [16,94,95]. Inhibiting LSD1 causes a loss in the mesenchymal signature in breast cancer cells, as well as in patient-derived circulating tumor cells, providing evidence for LSD1 as a potential therapeutic target for reversing the EMT process, particularly in CSCs. This has great potential and impact in reducing tumor recurrence not only by preventing the metastasis of BCSCs but also to eliminate the tumorigenic potential of CSCs. Unraveling the interplay between chromatin-associated kinases and SETDB1 in EMT and CSCs in breast metastases will be required in the future to enable the development of selective SETDB1 inhibitors. Furthermore, to address the enormous potential of SETDB1 targeting in breast cancer, combination studies combining SETDB1 drugs either alone or in combination with other classes of epigenetic drugs such as LSD1 inhibitors, either alone or with immunotherapy or chemotherapy drugs, are urgently required. In summary, development of specific SETDB1 inhibitors offers novel therapeutic strategies for metastatic breast cancer, where few options remain to date.

## 8. Conclusions

Exactly how histone modifications reprogram gene expression during metastasis is poorly understood. Here, we highlighted that the histone methyltransferase SETDB1 is responsible for the methylation of H3K9, which represses genes through heterochromatin formation. SETDB1 influences breast cancer progression in a number of ways such as through the induction and maintenance of stem cells, EMT programming, and metabolic control. SETDB1 also impacts a verity of cellular pathways that regulate gene expression in health and disease. SETDB1 is implicated in the formation of the cancer stem cells that give rise to metastases through a variety of mechanisms such as metabolic pathways and EMT. Therapies might need to be developed that target not only the cancer cells but also the aggressive stem cell compartment that promotes metastasis and therapeutic resistance. Further work is needed to fully establish SETDB1’s full biological repertoire and its role in cancer development. Given the presence of isoforms, it will also be important to develop drugs that target different regions and isoforms such as the NES/NLS regions that facilitate its nuclear and cytoplasmic levels. Furthermore, while we discussed the link of SETDB1 with protein kinases, we cannot exclude the possibility of histone methyltransferase working with phosphatases to regulate gene expression, as nuclear phosphatases were found to be present in breast cancer stem cells and circulating tumor cells [16,96]. A deeper understanding of the mechanism via which SETDB1 regulates expression of key genes in breast cancer will help us to develop a more targeted approach to finding an effective and selective inhibitor.

## Figures and Tables

**Table 1:** Known deleterious mutations of SETDB1.

Mutation	Location	Nucleotide Change AND Mutation Type	Effect
H1224K	C-terminal (SET domain)		Impaired Histone H3 (H3)-methylase activity Accelerated melanoma
C1226A	C-terminal (SET domain)		Impaired H3-methylase activity Accelerated melanoma
C1279Y	C-terminal (SET domain)		Impaired H3-methylase activity
Y249X	N-terminal	747 T > A Nonsense	Loss of function Multiple primary melanoma (MPM) development
V132FS	N-terminal	395–399del5 Frameshift	Loss of function Premature stop codon indicated in MPM development
G869E	C-terminal (Bifurcated SET)	2606 G > A Missense	
C911F	C-terminal (Bifurcated SET)	2732 G > T Missense	Unknown
S947C	C-terminal (Bifurcated SET)	2840 C > G Missense	Unknown
P226RFSX4 P226RFSX4	N-terminal N-terminal	677–693del17 Frameshift (Duplicate)	Loss of function Duplicate mutation
F1250DEL	C-terminal (Post-SET)	3747–3749del In-frame deletion	Unknown
K674SFSX73	C-terminal (Pre-SET-MBD aa sequence)	2020del A Frameshift	Loss of function

## References

[1] Ferlay J., Soerjomataram I., Dikshit R., Eser S., Mathers C., Rebelo M., Parkin D.M., Forman D., Bray F. (2015). Cancer incidence and mortality worldwide: Sources, methods and major patterns in GLOBOCAN 2012. Int. J. Cancer.

[2] Australian Institute of Health and Welfare (2019). Cancer Data in Australia.

[3] Filipova A., Seifrtova M., Mokry J., Dvorak J., Rezacova M., Filip S., Diaz-Garcia D. (2014). Breast cancer and cancer stem cells: a mini-review. Tumori.

[4] Wang H., Unternaehrer J.J. (2019). Epithelial-mesenchymal Transition and Cancer Stem Cells: At the Crossroads of Differentiation and Dedifferentiation. Dev Dyn.

[5] Thakur C., Chen F. (2019). Connections between metabolism and epigenetics in cancers. Semin. Cancer Biol..

[6] Ye X., Weinberg R.A. (2015). Epithelial–Mesenchymal Plasticity: A Central Regulator of Cancer Progression. Trends Cell Biol..

[7] Hanahan D., Weinberg R.A. (2011). Hallmarks of Cancer: The Next Generation. Cell.

[8] Collina F., Di Bonito M., Li Bergolis V., De Laurentiis M., Vitagliano C., Cerrone M., Nuzzo F., Cantile M., Botti G. (2015). Prognostic Value of Cancer Stem Cells Markers in Triple-Negative Breast Cancer. Biomed Res Int..

[9] Lee W.J., Kim S.C., Yoon J.H., Yoon S.J., Lim J., Kim Y.S., Kwon S.W., Park J.H. (2016). Meta-Analysis of Tumor Stem-Like Breast Cancer Cells Using Gene Set and Network Analysis. PLoS ONE.

[10] Ginestier C., Hur M.H., Charafe-Jauffret E., Monville F., Dutcher J., Brown M., Jacquemier J., Viens P., Kleer C.G., Liu S. (2007). ALDH1 is a marker of normal and malignant human mammary stem cells and a predictor of poor clinical outcome. Cell Stem Cell.

[11] Lin C.Y., Barry-Holson K.Q., Allison K.H. (2016). Breast cancer stem cells: are we ready to go from bench to bedside?. Histopathology.

[12] Jones P.A., Baylin S.B. (2007). The epigenomics of cancer. Cell.

[13] Klemm S.L., Shipony Z., Greenleaf W.J. (2019). Chromatin accessibility and the regulatory epigenome. Nat. Rev. Genet..

[14] Torres I.O., Fujimori D.G. (2015). Functional coupling between writers, erasers and readers of histone and DNA methylation. Curr. Opin. Struct. Biol..

[15] Muntean A.G., Hess J.L. (2009). Epigenetic dysregulation in cancer. Am. J. Pathol..

[16] Boulding T., McCuaig R.D., Tan A., Hardy K., Wu F., Dunn J., Kalimutho M., Sutton C.R., Forwood J.K., Bert A.G. (2018). LSD1 activation promotes inducible EMT programs and modulates the tumour microenvironment in breast cancer. Sci. Rep..

[17] Tam W.L., Weinberg R.A. (2013). The epigenetics of epithelial-mesenchymal plasticity in cancer. Nat. Med..

[18] Yang L., Xia L., Wu D.Y., Wang H., Chansky H.A., Schubach W.H., Hickstein D.D., Zhang Y. (2002). Molecular cloning of ESET, a novel histone H3-specific methyltransferase that interacts with ERG transcription factor. Oncogene.

[19] Ryu T.Y., Kim K., Kim S.-K., Oh J.-H., Min J.-K., Jung C.-R., Son M.-Y., Kim D.-S., Cho H.-S. (2019). SETDB1 regulates SMAD7 expression for breast cancer metastasis. BMB Rep..

[20] Suriyamurthy S., Baker D., Ten Dijke P., Iyengar P.V. (2019). Epigenetic Reprogramming of TGF-β Signaling in Breast Cancer. Cancers.

[21] Wu F., McCuaig D.R., Sutton R.C., Tan H.A., Jeelall Y., Bean G.E., Dai J., Prasanna T., Batham J., Malik L. (2019). Nuclear-Biased DUSP6 Expression is Associated with Cancer Spreading Including Brain Metastasis in Triple-Negative Breast Cancer. Int. J. Mol. Sci..

[22] Wozniak G.G., Strahl B.D. (2014). Hitting the ‘mark’: Interpreting lysine methylation in the context of active transcription. Biochim. Biophys. Acta (BBA) Gene Regul. Mech..

[23] Rose N.R., Klose R.J. (2014). Understanding the relationship between DNA methylation and histone lysine methylation. Biochim. Biophys. Acta (BBA) Gene Regul. Mech..

[24] Li H., Rauch T., Chen Z.-X., Szabó P.E., Riggs A.D., Pfeifer G.P. (2006). The Histone Methyltransferase SETDB1 and the DNA Methyltransferase DNMT3A Interact Directly and Localize to Promoters Silenced in Cancer Cells. J. Biol. Chem..

[25] Kaniskan H.Ü., Konze K.D., Jin J. (2015). Selective inhibitors of protein methyltransferases. J. Med. Chem..

[26] Chen K., Zhang F., Ding J., Liang Y., Zhan Z., Zhan Y., Chen L.-H., Ding Y. (2017). Histone Methyltransferase SETDB1 Promotes the Progression of Colorectal Cancer by Inhibiting the Expression of TP53. J. Cancer.

[27] Schultz D.C., Ayyanathan K., Negorev D., Maul G.G., Rauscher F.J. (2002). SETDB1: A novel KAP-1-associated histone H3, lysine 9-specific methyltransferase that contributes to HP1-mediated silencing of euchromatic genes by KRAB zinc-finger proteins. Genes Dev..

[28] Xu M., Gonzalez-Hurtado E., Martinez E. (2016). Core promoter-specific gene regulation: TATA box selectivity and Initiator-dependent bi-directionality of serum response factor-activated transcription. Biochim. Biophys. Acta.

[29] Romier C., Cocchiarella F., Mantovani R., Moras D. (2003). The NF-YB/NF-YC Structure Gives Insight into DNA Binding and Transcription Regulation by CCAAT Factor NF-Y. J. Biol. Chem..

[30] Blackburn M.L., Chansky H.A., Zielinska-Kwiatkowska A., Matsui Y., Yang L. (2003). Genomic structure and expression of the mouse ESET gene encoding an ERG-associated histone methyltransferase with a SET domain. Biochim. Biophys. Acta (BBA) Gene Struct. Expr..

[31] Cho S., Park J.S., Kang Y.-K. (2013). Regulated nuclear entry of over-expressed Setdb1. Genes Cells.

[32] Yeap L.-S., Hayashi K., Surani M.A. (2009). ERG-associated protein with SET domain (ESET)-Oct4 interaction regulates pluripotency and represses the trophectoderm lineage. Epigenetics Chromatin.

[33] Loyola A., Tagami H., Bonaldi T., Roche D., Quivy J.P., Imhof A., Nakatani Y., Dent S.Y.R., Almouzni G. (2009). The HP1alpha-CAF1-SetDB1-containing complex provides H3K9me1 for Suv39-mediated K9me3 in pericentric heterochromatin. EMBO Rep..

[34] Kang Y.-K. (2014). SETDB1 in Early Embryos and Embryonic Stem Cells. Curr. Issues Mol. Biol..

[35] Parikh K., Cang S., Sekhri A., Liu D. (2014). Selective inhibitors of nuclear export (SINE)—A novel class of anti-cancer agents. J. Hematol. Oncol..

[36] Cho S., Park J.S., Kang Y.-K. (2011). Dual functions of histone-lysine N-methyltransferase Setdb1 protein at promyelocytic leukemia-nuclear body (PML-NB): maintaining PML-NB structure and regulating the expression of its associated genes. J. Biol. Chem..

[37] Fujita N., Watanabe S., Ichimura T., Ohkuma Y., Chiba T., Saya H., Nakao M. (2003). MCAF Mediates MBD1-Dependent Transcriptional Repression. Mol. Cell. Biol..

[38] Basavapathruni A., Gureasko J., Porter Scott M., Hermans W., Godbole A., Leland P.A., Boriack-Sjodin P.A., Wigle T.J., Copeland R.A., Riera T.V. (2016). Characterization of the Enzymatic Activity of SETDB1 and Its 1:1 Complex with ATF7IP. Biochemistry.

[39] Timms R.T., Tchasovnikarova I.A., Antrobus R., Dougan G., Lehner P.J. (2016). ATF7IP-Mediated Stabilization of the Histone Methyltransferase SETDB1 Is Essential for Heterochromatin Formation by the HUSH Complex. Cell Rep..

[40] Yang L., Mei Q., Zielinska-Kwiatkowska A., Matsui Y., Blackburn M.L., Benedetti D., Krumm A.A., Taborsky G.J., Chansky H.A. (2003). An ERG (ets-related gene)-associated histone methyltransferase interacts with histone deacetylases 1/2 and transcription co-repressors mSin3A/B. Biochem. J..

[41] Lallemand-Breitenbach V., de Thé H. (2010). PML nuclear bodies. Cold Spring Harb. Perspect. Biol..

[42] Dellaire G., Bazett-Jones D.P. (2004). PML nuclear bodies: dynamic sensors of DNA damage and cellular stress. BioEssays.

[43] Bohan C.A., Kashanchi F., Ensoli B., Buonaguro L., Boris-Lawrie K.A., Brady J.N. (2018). Analysis of Tat transactivation of human immunodeficiency virus transcription in vitro. Gene Expr..

[44] Poletti V., Mavilio F. (2017). Interactions between Retroviruses and the Host Cell Genome. Mol. Ther. Methods Clin. Dev..

[45] Karanth A.V., Maniswami R.R., Prashanth S., Govindaraj H., Padmavathy R., Jegatheesan S.K., Mullangi R., Rajagopal S. (2017). Emerging role of SETDB1 as a therapeutic target. Expert Opin. Ther. Targets.

[46] Keniry A., Gearing L.J., Jansz N., Liu J., Holik A.Z., Hickey P.F., Kinkel S.A., Moore D.L., Breslin K., Chen K. (2016). Setdb1-mediated H3K9 methylation is enriched on the inactive X and plays a role in its epigenetic silencing. Epigenetics Chromatin.

[47] Herz H.-M., Garruss A., Shilatifard A. (2013). SET for life: biochemical activities and biological functions of SET domain-containing proteins. Trends Biochem. Sci..

[48] Ryan R.F., Schultz D.C., Ayyanathan K., Singh P.B., Friedman J.R., Fredericks W.J., Rauscher F.J. (1999). KAP-1 corepressor protein interacts and colocalizes with heterochromatic and euchromatic HP1 proteins: a potential role for Krüppel-associated box-zinc finger proteins in heterochromatin-mediated gene silencing. Cell. Biol..

[49] Wiznerowicz M., Jakobsson J., Szulc J., Liao S., Quazzola A., Beermann F., Aebischer P., Trono D. (2007). The Krüppel-associated Box Repressor Domain Can Trigger de Novo Promoter Methylation during Mouse Early Embryogenesis. J. Biol. Chem..

[50] Ecco G., Imbeault M., Trono D. (2017). KRAB zinc finger proteins. Development.

[51] Rowe H.M., Kapopoulou A., Corsinotti A., Fasching L., Macfarlan T.S., Tarabay Y., Viville S., Jakobsson J., Pfaff S.L., Trono D. (2013). TRIM28 repression of retrotransposon-based enhancers is necessary to preserve transcriptional dynamics in embryonic stem cells. Genome Res..

[52] Dodge J.E., Kang Y.-K., Beppu H., Lei H., Li E. (2004). Histone H3-K9 methyltransferase ESET is essential for early development. Mol. Cell. Biol..

[53] Karimi M.M., Goyal P., Maksakova I.A., Bilenky M., Leung D., Tang J.X., Shinkai Y., Mager D.L., Jones S., Hirst M. (2011). DNA methylation and SETDB1/H3K9me3 regulate predominantly distinct sets of genes, retroelements, and chimeric transcripts in mESCs. Cell Stem Cell.

[54] Tan S.-L., Nishi M., Ohtsuka T., Matsui T., Takemoto K., Kamio-Miura A., Aburatani H., Shinkai Y., Kageyama R. (2012). Essential roles of the histone methyltransferase ESET in the epigenetic control of neural progenitor cells during development. Development.

[55] Eymery A., Liu Z., Ozonov E.A., Stadler M.B., Peters A.H.F.M. (2016). The methyltransferase Setdb1 is essential for meiosis and mitosis in mouse oocytes and early embryos. Development.

[56] Adoue V., Binet B., Malbec A., Fourquet J., Romagnoli P., van Meerwijk J.P.M., Amigorena S., Joffre O.P. (2019). The Histone Methyltransferase SETDB1 Controls T Helper Cell Lineage Integrity by Repressing Endogenous Retroviruses. Immunity.

[57] Takikita S., Muro R., Takai T., Otsubo T., Kawamura Y.I., Dohi T., Oda H., Kitajima M., Oshima K., Hattori M. (2016). A Histone Methyltransferase ESET Is Critical for T Cell Development. J. Immunol..

[58] Melssen M., Slingluff C.L. (2017). Vaccines targeting helper T cells for cancer immunotherapy. Curr. Opin. Immunol..

[59] Sun Q.-Y., Ding L.-W., Xiao J.-F., Chien W., Lim S.-L., Hattori N., Goodglick L., Chia D., Mah V., Alavi M. (2015). SETDB1 accelerates tumourigenesis by regulating the WNT signalling pathway. J. Pathol..

[60] Kang H.C., Kim H.K., Lee S., Mendez P., Kim J.W., Woodard G., Yoon J.-H., Jen K.-Y., Fang L.T., Jones K. (2016). Whole exome and targeted deep sequencing identify genome-wide allelic loss and frequent SETDB1 mutations in malignant pleural mesotheliomas. Oncotarget.

[61] Song G., Ouyang G., Bao S. (2005). The activation of Akt/PKB signaling pathway and cell survival. J. Cell. Mol. Med..

[62] Wang J., Xu-Monette Z.Y., Jabbar K.J., Shen Q., Manyam G.C., Tzankov A., Visco C., Wang J., Montes-Moreno S., Dybkær K. (2017). AKT Hyperactivation and the Potential of AKT-Targeted Therapy in Diffuse Large B-Cell Lymphoma. Am. J. Pathol..

[63] Wang G., Long J., Gao Y., Zhang W., Han F., Xu C., Sun L., Yang S.-C., Lan J., Hou Z. (2019). SETDB1-mediated methylation of Akt promotes its K63-linked ubiquitination and activation leading to tumorigenesis. Nat. Cell Biol..

[64] Guo J., Dai X., Laurent B., Zheng N., Gan W., Zhang J., Guo A., Yuan M., Liu P., Asara J.M. (2019). AKT methylation by SETDB1 promotes AKT kinase activity and oncogenic functions. Nat. Cell Biol..

[65] Zhang H., Cai K., Wang J., Wang X., Cheng K., Shi F., Jiang L., Zhang Y., Dou J. (2014). MiR-7, Inhibited Indirectly by LincRNA HOTAIR, Directly Inhibits SETDB1 and Reverses the EMT of Breast Cancer Stem Cells by Downregulating the STAT3 Pathway. Stem Cells.

[66] Rodriguez-Paredes M., Martinez de Paz A., Simó-Riudalbas L., Sayols S., Moutinho C., Moran S., Villanueva A., Vázquez-Cedeira M., Lazo P.A., Carneiro F. (2014). Gene amplification of the histone methyltransferase SETDB1 contributes to human lung tumorigenesis. Oncogene.

[67] Xu X., Zheng L., Yuan Q., Zhen G., Crane J.L., Zhou X., Cao X. (2018). Transforming growth factor-β in stem cells and tissue homeostasis. Bone Res..

[68] Wu M., Fan B., Guo Q., Li Y., Chen R., Lv N., Diao Y., Luo Y. (2018). Knockdown of SETDB1 inhibits breast cancer progression by miR-381-3p-related regulation. Bone Res..

[69] Du D., Katsuno Y., Meyer D., Budi E.H., Chen S.-H., Koeppen H., Wang H., Akhurst R.J., Derynck R. (2018). Smad3-mediated recruitment of the methyltransferase SETDB1/ESET controls Snail1 expression and epithelial-mesenchymal transition. EMBO Rep..

[70] Iglesias J.M., Gumuzio J., Martin A.G. (2017). Linking Pluripotency Reprogramming and Cancer. Stem Cells Transl. Med..

[71] Miyagi S., Koide S., Saraya A., Wendt G.R., Oshima M., Konuma T., Yamazaki S., Mochizuki-Kashio M., Nakajima-Takagi Y., Wang C. (2014). The TIF1β-HP1 system maintains transcriptional integrity of hematopoietic stem cells. Stem Cell Rep..

[72] Ugarte F., Sousae R., Cinquin B., Martin E.W., Krietsch J., Sanchez G., Inman M., Tsang H., Warr M., Passegué E. (2015). Progressive Chromatin Condensation and H3K9 Methylation Regulate the Differentiation of Embryonic and Hematopoietic Stem Cells. Stem Cell Rep..

[73] Chakravarty S., Pathak S.S., Maitra S., Khandelwal N., Chandra K.B., Kumar A., Pandey S.C. (2014). Chapter Four - Epigenetic Regulatory Mechanisms in Stress-Induced Behavior. International Review of Neurobiology.

[74] Lehnertz B., Pabst C., Su L., Miller M., Liu F., Yi L., Zhang R., Krosl J., Yung E., Kirschner J. (2014). The methyltransferase G9a regulates HoxA9-dependent transcription in AML. Genes Dev..

[75] Koide S., Oshima M., Takubo K., Yamazaki S., Nitta E., Saraya A., Aoyama K., Kato Y., Miyagi S., Nakajima-Takagi Y. (2016). Setdb1 maintains hematopoietic stem and progenitor cells by restricting the ectopic activation of nonhematopoietic genes. Blood.

[76] Vander Heiden M.G., Cantley L.C., Thompson C.B. (2009). Understanding the Warburg effect: the metabolic requirements of cell proliferation. Science.

[77] Warburg O., Wind F., Negelein E. (1927). THE METABOLISM OF TUMORS IN THE BODY. J. Gen Physiol.

[78] Dong C., Yuan T., Wu Y., Wang Y., Fan T.W.M., Miriyala S., Lin Y., Yao J., Shi J., Kang T. (2013). Loss of FBP1 by Snail-mediated repression provides metabolic advantages in basal-like breast cancer. Cancer Cell.

[79] Luqmani Y.A. (2005). Mechanisms of Drug Resistance in Cancer Chemotherapy. Med Princ. Pract..

[80] Yang M.-H., Imrali A., Heeschen C. (2015). Circulating cancer stem cells: the importance to select. Chin. J. Cancer Res..

[81] Chen W., Dong J., Haiech J., Kilhoffer M.-C., Zeniou M. (2016). Cancer Stem Cell Quiescence and Plasticity as Major Challenges in Cancer Therapy. Stem Cells Int..

[82] Song Y., Wu F., Wu J. (2016). Targeting histone methylation for cancer therapy: enzymes, inhibitors, biological activity and perspectives. J. Hematol. Oncol..

[83] Lee J.-K., Kim K.-C. (2013). DZNep, inhibitor of S-adenosylhomocysteine hydrolase, down-regulates expression of SETDB1 H3K9me3 HMTase in human lung cancer cells. Biochem. Biophys. Res. Commun..

[84] Ryu H., Lee J., Hagerty S.W., Soh B.Y., McAlpin S.E., Cormier K.A., Smith K.M., Ferrante R.J. (2006). ESET/SETDB1 gene expression and histone H3 (K9) trimethylation in Huntington’s disease. Proc. Natl. Acad. Sci. USA.

[85] Noh H.-J., Kim K.-A., Kim K.-C. (2014). p53 Down-regulates SETDB1 gene expression during paclitaxel induced-cell death. Biochem. Biophys. Res. Commun..

[86] Ho J.C., Abdullah L.N., Pang Q.Y., Jha S., Chow E.K.-H., Yang H., Kato H., Poellinger L., Ueda J., Lee K.L. (2017). Inhibition of the H3K9 methyltransferase G9A attenuates oncogenicity and activates the hypoxia signaling pathway. PLoS ONE.

[87] Chan D.A., Giaccia A.J. (2007). Hypoxia, gene expression, and metastasis. Cancer Metastasis Rev..

[88] Fiskus W., Rao R., Balusu R., Ganguly S., Tao J., Sotomayor E., Mudunuru U., Smith J.E., Hembruff S.L., Atadja P. (2012). Superior Efficacy of a Combined Epigenetic Therapy against Human Mantle Cell Lymphoma Cells. Cancer Res..

[89] Fiskus W., Wang Y., Sreekumar A., Buckley K.M., Shi H., Jillella A., Ustun C., Rao R., Fernandez P., Chen J. (2009). Combined epigenetic therapy with the histone methyltransferase EZH2 inhibitor 3-deazaneplanocin A and the histone deacetylase inhibitor panobinostat against human AML cells. Blood.

[90] Chiba T., Suzuki E., Negishi M., Saraya A., Miyagi S., Konuma T., Tanaka S., Tada M., Kanai F., Imazeki F. (2012). 3-Deazaneplanocin A is a promising therapeutic agent for the eradication of tumor-initiating hepatocellular carcinoma cells. Int. J. Cancer.

[91] Kampan N.C., Madondo M.T., McNally O.M., Quinn M., Plebanski M. (2015). Paclitaxel and Its Evolving Role in the Management of Ovarian Cancer. Biomed Res. Int..

[92] Safe S., Abbruzzese J., Abdelrahim M., Hedrick E. (2018). Specificity Protein Transcription Factors and Cancer: Opportunities for Drug Development. Cancer Prev. Res..

[93] Xing Y., Lin N.U., Maurer M.A., Chen H., Mahvash A., Sahin A., Akcakanat A., Li Y., Abramson V., Litton J. (2019). Phase II trial of AKT inhibitor MK-2206 in patients with advanced breast cancer who have tumors with PIK3CA or AKT mutations, and/or PTEN loss/PTEN mutation. Breast Cancer Res. BCR.

[94] Sutcliffe E.L., Bunting K.L., He Y.Q., Li J., Phetsouphanh C., Seddiki N., Zafar A., Hindmarsh E.J., Parish C.R., Kelleher A.D. (2011). Chromatin-associated protein kinase C-θ regulates an inducible gene expression program and microRNAs in human T lymphocytes. Mol. Cell.

[95] Zafar A., Wu F., Hardy K., Li J., Tu W.J., McCuaig R., Harris J., Khanna K.K., Attema J., Gregory P.A. (2014). Chromatinized protein kinase C-θ directly regulates inducible genes in epithelial to mesenchymal transition and breast cancer stem cells. Mol. Cell Biol..

[96] Boulding T., Wu F., McCuaig R., Dunn J., Sutton C.R., Hardy K., Tu W., Bullman A., Yip D., Dahlstrom J.E. (2016). Differential Roles for DUSP Family Members in Epithelial-to-Mesenchymal Transition and Cancer Stem Cell Regulation in Breast Cancer. PLoS ONE.

